# Heart rate thresholds for cardiovascular risk and sympathetic activation in the metabolic syndrome

**DOI:** 10.1007/s00592-022-01945-5

**Published:** 2022-07-29

**Authors:** Gino Seravalle, Jennifer Vanoli, Concetta Molisano, Valeria Merati, Guido Grassi

**Affiliations:** Clinica Medica, Department of Medicine and Surgery, University of Milano-Bicocca, Via Pergolesi 33, 20052 Monza, Italy

**Keywords:** Heart rate, Sympathetic nerve traffic, Norepinephrine, Metabolic syndrome, Cardiovascular risk

## Abstract

**Aims:**

We examined whether to what extent resting heart rate (HR) values are capable to reflect in the metabolic syndrome (MS) a different degree of sympathetic activation. We also thought to determine at which HR cutoff values the sympathetic nervous system becomes more activated in the MS.

**Methods:**

In 70 MS patients aged 55.5 ± 1.8 (mean ± SEM) years we evaluated muscle sympathetic nerve traffic (MSNA, microneurography) and venous plasma norepinephrine (NE, HPLC assay), subdividing the study population in three different subgroups according to resting clinic and 24-h HR values (< 70, 70–79 and ≥ 80 beats/min).

**Results:**

MS patients with clinic HR values ≥ 80 beats/min displayed MSNA and NE values significantly increased when compared to those found in MS with HR between 70 and 79 beats/min or below 70 beats/min (MSNA: 55.2 ± 0.9 vs 44.6 ± 0.6 and 39.2 ± 0.6 bursts/min, *P* < 0.01, NE: 403.9 ± 6.9 vs 330.1 ± 4.3 and 258.3 ± 6.8 pg/ml, respectively, *P* < 0.01). A similar behavior was observed for 24-h HR. In the group as a whole both MSNA and plasma NE showed highly significant direct relationships with clinic HR, the correlation being similar for MSNA and NE (*r* = 0.89 and *r* = 0.91, *P* < 0.01 for both) Similar significant relationships were also found between 24-h HR values and MSNA or NE.

**Conclusions:**

In the MS HR values ≥ 80 beats/min are associated with an increased sympathetic activation, both when assessed by direct recording of MSNA and when evaluated as plasma NE. The sympathetic overdrive parallels for magnitude the HR elevations, this being the case for both clinic and 24-h HR.

## Introduction

Studies performed in the past two decades have shown that a marked activation of the neuroadrenergic influences to the heart and the peripheral circulation characterizes the metabolic syndrome (MS) [1–3]. This has been documented by employing different methodologies to assess sympathetic neural function, such as (1) the assay of urinary excretion of catecholamine metabolites or circulating venous plasma levels of the adrenergic neurotransmitter norepinephrine (NE) [4, 5], (2) the power spectral analysis of the spontaneous oscillations of heart rate (HR) in specific temporal windows [6], (3) the assessment of the whole-body NE kinetics [7, 8] and (4) the direct microneurographic recording of efferent postganglionic muscle sympathetic nerve traffic (MSNA) [3, 7–10]. A further approach, based on evaluation of HR as sympathetic biomarker, has also been shown to represent in some of the different clinical conditions clustering in the metabolic syndrome, such as obesity, prediabetes and hypertension, a variable with important prognostic value capable to predict, independently on other confounders, the development of fatal and non-fatal cardiovascular outcomes [11–14]. Specifically, HR > 80 beats/min and 73 beats/min have been identified as cutoff values above which cardiovascular risk is elevated in hypertension and prediabetes, respectively [13, 14], and appropriate cardiovascular drug treatment with HR lowering effects should be considered. However, no information is so far available on whether the sympathetic nervous system is differently activated in MS patients displaying resting HR values above or below the above-mentioned cutoffs. This information is crucial in order to determine whether more or less elevated HR values are capable to reflect in the MS a different degree of sympathetic activation. It is also undefined at which HR cutoff value the sympathetic nervous system becomes more activated in the MS. Both information is of clinical relevance in order to determine the most appropriate pharmacological intervention. The present study was designed to address this issue by assessing sympathetic cardiovascular drive in a large sample of MS patients displaying resting HR values at different cutoffs. This was done by assessing sympathetic cardiovascular drive via venous plasma NE assay and microneurographic recording of MSNA. The assessment of HR included not only clinic but also 24-h values, allowing to relate static and dynamic figures of this hemodynamic variable to the above-mentioned neuroadrenergic markers.

## Methods

### Population

The study population consisted of 70 patients of both genders (60 males, 10 females) with an age range between 42 and 61 years. For all patients the evaluation was done retrospectively and it was based on the detection of an HR value below or above 70 beats/min at the office visit performed the day preceding the microneurographic nerve traffic recording session made in the frame of different investigations carried out between 2014 and 2021. All individuals included in the study were in sinus rhythm, and no subject had a history of myocardial infarction in the 12 months before the study or clinical or laboratory evidence of valvular heart disease, congestive heart failure, thyroid dysfunction, overt diabetes mellitus, secondary hypertension, renal insufficiency, obstructive sleep apnea, or any other condition known to affect autonomic modulation of the cardiovascular system [15]. Recruited patients were affected by the MS, displaying at least three of the five diagnostic criteria for the disease proposed by the National Cholesterol Educational Program Adult Treatment Panel in the 2005 revised version [16], that is waist circumference more than 102 cm in men and more than 88 cm in women; fasting triglyceride level at least 1.69 mmol/l; HDL-cholesterol less than 1.04 mmol/l in men and less than 1.29 mmol/l in women; blood pressure (BP) at least 130/85 mmHg; and fasting glucose level at least 6.1 mmol/l. None of the 70 patients diagnosed as having the MS was under pharmacological treatment because the diagnosis was of a new onset. Patients were evaluated on an outpatient basis and gave their written consent to the study after being informed of its nature and purpose. The study protocol was approved by the Ethics Committee of one of the institutions involved.

### Measurements

In all patients, BP was measured by a mercury sphygmomanometer, taking the first and fifth Korotkoff sounds to identify systolic and diastolic BP values, respectively, and by a finger photoplethysmographic device (Finapres 2300, Englewood, Colorado, USA) capable of providing accurate and reproducible beat-to-beat BP values [3, 10]. Waist and hip circumferences were determined using a non-stretchable tape. Waist measurements were taken at the natural waist midpoint between the lowest edge of the rib cage and the highest point of the iliac crest. Hip circumference was measured at the point of maximum circumference over the buttocks. Body mass index was obtained by dividing body weight in kilograms by the square of the height in meters. Measurements also included fasting plasma glucose (radioenzymatic method) and insulin (radioimmunoassay) levels in the fasting state, which allowed us to estimate insulin resistance by the homeostasis model assessment (HOMA). The HOMA index was calculated according to the formula fasting plasma insulin × fasting plasma glucose/22.5 [17]. The same blood sample was used also to assess other metabolic variables, including plasma total cholesterol (TC), HDL-cholesterol, LDL-cholesterol and triglycerides (enzymatic method). HR was monitored beat-to-beat by a standard EKG lead. Venous plasma NE concentration was measured via high-performance liquid chromatography with electrochemical detection (Machery-Nagel ET 200/4 Nucleosil 100-5 C18 column, Machery-Nagel, and Waters 460 electrochemical detector; Waters GmbH, Eschborn, Germany) [18] in blood withdrawn from an antecubital vein of the arm not used for BP measurements. Measurements also included (a) MSNA via the microneurographic technique [3, 7–10], (b) respiration rate via a pneumotacograph and (c) echocardiographic assessment of the end-diastolic and end-systolic left ventricular internal diameters, interventricular septum thickness and posterior wall thickness, left atrial diameters and left ventricular ejection fraction, measured from the four-chamber apical projection using the product area times length [19, 20]. Echocardiographic data also included mitral flow [early diastolic peak flow velocity (E wave) and late diastolic peak flow velocity (A wave)] and flow at the left ventricular outflow tract values. Left ventricular mass index was calculated by the Devereux formula and normalized to body surface area [20]. An EKG-Holter monitoring was performed during the 24-h period in the days preceding the evaluation of the sympathetic neural function.

Simultaneous MSNA, beat-to-beat HR and BP recordings were digitized with a sampling frequency of 1000 Hz (PowerLab Recording System Model ML870 8/30; AD Instruments, Bella Vista, New South Wales, Australia). MSNA was quantified over a 30-min period as bursts incidence over time (bursts/min) [3, 7–10]. This quantification has been shown to be highly reproducible, that is to differ by only 4.3% when assessed on two separate occasions [21].

### Protocol and data analysis

All participants were examined in the morning after a light breakfast and an overnight abstinence from alcohol and coffee consumption. They were asked to assume the supine position, after which 3 sphygmomanometric BP and HR (palpatory method, radial artery) were obtained. Following the BP and HR measurements, the patients were fitted with an intravenous cannula and the devices to measure finger BP and to record an EKG. Blood samples were taken 30 min after positioning the venous cannula. A microelectrode was then inserted into a peroneal nerve to obtain MSNA, which was recorded together with finger BP and the EKG during a 30-min period. Data were collected in a semi-dark and quiet room kept at a constant temperature of 20°–22 °C. As mentioned above, the study had a retrospective nature and included data collected and already blindly analyzed for other microneurographic studies not related to the objective of the present investigation. This allowed to avoid any potential bias in data analysis. Values from individual participants were averaged (see below) and expressed as means ± SEM. The study population was subdivided into three different groups according to the clinic HR values displayed at rest by the patients. Comparisons between groups were made by two-way ANOVA, using the Student *t* test for unpaired observations or by Chi-square statistic to determine their differences. The Pearson correlation coefficient was used to determine the relationships between HR, MSNA, NE and other parameters, a *P* < 0.05 being taken as the minimal level of statistical significance. All statistical analyses were performed by SAS software version 9.4 (SAS Institute Inc., Cary, NC, USA).

## Results

As shown in Table 1 the three groups of MS patients characterized by resting clinic HR values below 70, between 70 and 79, and above 80 beats/min displayed similar gender distribution and superimposable age. No significant difference between groups was found as far as body mass index, systolic and diastolic BP, serum glucose, plasma creatinine are concerned. Left ventricular ejection fraction was similar in the three groups, which also showed similar E/A values and almost superimposable left ventricular mass index.

**Table 1:** Demographic, hemodynamic and clinical characteristics of patients with the metabolic syndrome classified in three groups accordingly to different clinic heart rate (HR) values

Variable	HR < 70 b/min (*n* = 25)	HR 70–79 b/min (*n* = 25)	HR ≥ 80 b/min (*n* = 20)
Age (years)	55.8 ± 2.4	56.3 ± 2.0	54.7 ± 2.5
Gender (M/F, no.)	20/5	22/3	18/2
BMI (kg/m^2^)	30.2 ± 1.1	30.0 ± 1.2	31.7 ± 1.4
Clinic SBP (mmHg)	144.8 ± 3.2	142.0 ± 2.9	147.5 ± 3.8
Clinic DBP (mmHg) Clinic HR (b/min) 24-h HR (b/min)	92.1 ± 2.8 65.0 ± 0.8 63.6 ± 0.8	91.4 ± 2.7 75.2 ± 0.8 73.9 ± 0.6	93.7 ± 23.3 87.1 ± 1.3 85.8 ± 1.1
LVEF (%) E/A ratio (a.u.) LVMI (g/m^2^)	60.4 ± 1.3 1.10 ± 0.2 108.9 ± 3.3	59.6 ± 1.4 1.12 ± 0.2 111.1 ± 3.2	61.1 ± 1.9 1.11 ± 0.3 110.4 ± 3.8
Total cholesterol (mg/dl) HDL cholesterol (mg/dl) LDL cholesterol (md/dl) Triglycerides (mg/dl)	181.2 ± 5.1 35.4 ± 2.1 128.5 ± 4.9 150.5 ± 6.1	179.4 ± 4.4 36.9 ± 2.3 130.2 ± 4.4 148.6 ± 5.9	186.7 ± 5.9 37.7 ± 2.9 127.6 ± 5.3 155.4 ± 7.3
Serum glucose (mg/dl) Insulin (mU/l) HOMA.IR index (a.u.)	103.6 ± 2.4 13.5 ± 1.4 3.38 ± 0.2	104.8 ± 2.3 14.1 ± 1.3 3.51 ± 0.2	106.3 ± 2.9 13.9 ± 0.3 3.55 ± 0.3
Creatinine (mg/dl)	1.2 ± 0.2	0.99 ± 0.2	1.1 ± o.3
Respiration rate (breaths/min)	16.3 ± 1.3	17.2 ± 01.0	16.7 ± 1.5

Data are shown as means SEM

*M* males, *F* females, *BMI* body mass index, *SBP* systolic blood pressure, *DBP* diastolic blood pressure, *HR* heart rate, *LVEF* left ventricular ejection fraction, *LVMI* left ventricular mass index, *a.u.* arbitrary units, *HDL* high density lipoprotein, *LDL* low density lipoprotein, *HOMA-IR* homeostasis model assessment of insulin resistance

**Fig. 1:**
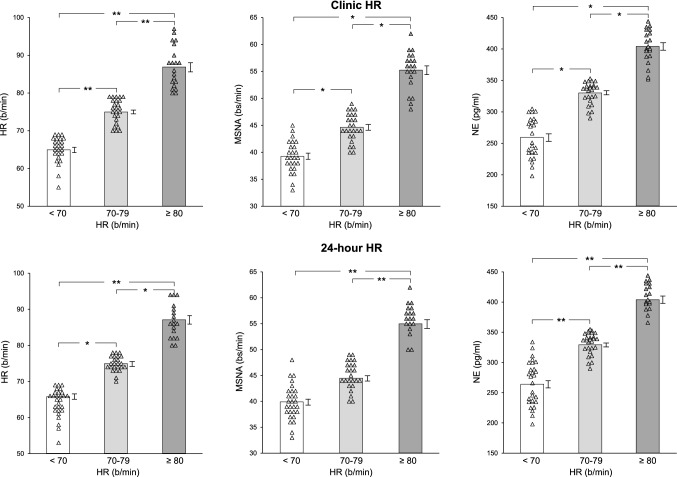
Upper panels: individual and average values (± SEM) of clinic heart rate (HR), muscle sympathetic nerve traffic (MSNA), and venous plasma norepinephrine (NE) in the groups of patients with the metabolic syndrome with resting clinic HR < 70 beats/min, between 70 and 79 beats/min, and ≥ 80 beats/min. Lower panels: Individual and average values (± SEM) of 24-h (24 h) heart rate (HR), muscle sympathetic nerve traffic (MSNA), and venous plasma norepinephrine (NE) in the groups of patients with the metabolic syndrome with resting 24-h HR < 70 beats min, between 70 and 79 beats/min, and ≥ 80 beats/min. Bs/min indicates bursts/min. Asterisks (*P < 0.05, **P < 0.01) refer to the statistical significance between groups

Individual and average clinic HR values assessed in the three MS groups are shown in Fig. 1, which also displays individual and average data obtained for 24-h HR monitoring, as well as the corresponding individual and average MSNA and venous plasma NE values. As expected, in MS patients resting HR showed values progressively and significantly greater from the group with an HR below 70 beats/min to the ones displaying HR between 70 and 79 and above 80 beats/min (Fig. 1, left upper panel). This was the case also for 24-h HR values (Fig. 1, left lower panel). More importantly, although consistent inter-individual differences were found, patients with clinic HR values greater than 80 beats/min displayed MSNA and NE values significantly increased when compared to those found in MS with HR between 70 and 79 beats/min or below 70 beats/min (Fig. 1, upper middle and right panels). A similar behavior was observed when the 24-h HR data were examined (Fig. 1, lower middle and right panels).

In the group as a whole both MSNA and plasma NE showed highly significant direct relationships with clinic HR, the correlation being similar for MSNA and NE (*r* = 0.896 and *r* = 0.890, respectively, *P* < 0.0001 for both). Similar significant relationships were also found between 24-h HR values and MSNA or NE (*r* = 0.918 and 0.913, respectively, *P* < 0.0001 for both). However, when the same relationships were sought separately in the 3 different groups of MS patients significant correlations were found in the groups displaying HR values between 70 and 79 and greater than 80 beats/min (data not shown). In the whole study population MSNA was significantly and directly related to clinic BP (*r* = 0.24 and *r* = 0.26, respectively, for systolic and diastolic BP, *P* < 0.05 for both), body mass index (*r* = 0.32, *P* < 0.01), waist circumference (*r* = 0.32, *P* < 0.01), waist-to-hip ratio (*r* = 0.38, *P* < 0.001), left ventricular mass index (*r* = 0.29, *P* < 0.05), plasma insulin (*r* = 0.33, *P* < 0.01) and HOMA index (*r* = 0.36, *P* < 0.01). Similar although less significant correlations were found between clinic HR, 24-h HR or plasma NE and the above-mentioned variables (data not shown). No significant correlation was found between the above-mentioned sympathetic biomarkers and serum triglycerides, serum total cholesterol, LDL and HDL cholesterol values.

## Discussion

Our study aimed to provide information on whether and to what extent HR values are capable to reflect in the MS a different degree of sympathetic activation. It is was also aimed at defining at which HR cutoff value the sympathetic nervous system becomes more activated in the MS. A qualifying feature of the present study was the assessment of the sympathetic neural function via the gold standard methodologies at present available in humans to directly evaluate neuroadrenergic cardiovascular drive, namely direct recording of MSNA and biochemical assay of venous plasma NE [15]. The results show that: (1) in patients with MS clinic HR values above 80 beats/min are accompanied by a sympathetic activation significantly and markedly greater for magnitude (about + 25–30%) than the one seen in MS patients with HR less than 80 beats/min, (2) this result is detectable when sympathetic activity is assessed either via the microneurographic technique or via the assay of venous plasma NE concentrations, (3) the above-mentioned HR cutoff value is valid not only for clinic HR as assessed via the palpatory method of the radial artery, but also for 24 h HR assessed via the Holter monitoring technique, (4) the assessment of the adrenergic cardiovascular drive was in MS superimposable for sensitivity both when the NE assay and the MSNA recording approach was applied, at variance from what we found in other clinical conditions in which we performed the same evaluation [22, 23]. Taken together these findings allow to conclude that in the MS, as it has been previously reported by our group in essential hypertension [22], a cutoff HR value amounting to 80 betas/min allows to detect the occurrence of a hyperadrenergic state which affects the cardiovascular system as a whole. They additionally document that a lower HR cutoff, such as the one reported for another component of the MS, namely glucose intolerance or prediabetes, appears to be less capable to fully reflect the adrenergic overdrive characterizing this clinical condition. Identification of cutoff HR values reflecting greater sympathetic activation has pathophysiological and clinical relevance considering that a high sympathetic cardiovascular drive is associated in a number of cardiovascular disease, such as stroke, chronic kidney disease and chronic heart failure, with an elevated risk of fatal and non-fatal cardiovascular events, independently on other confounders [24–26]. In addition in several cardiometabolic disease, including MS and obesity, increased cardiovascular influences are involved in favoring the development of metabolic disarray, such as glucose intolerance and insulin resistance [27–29].

Two other results of our study deserve to be briefly discussed. The first one refers to the question of whether or not definition of the specific HR cutoff for the MS associated with greater sympathoexcitation appears to be specific for this condition or rather common to other clinical states characterized by an adrenergic overdrive. Indeed, while the same HR cutoff value was detected in hypertension and chronic kidney disease, a lower one characterizes chronic heart failure, suggesting that no generalization of the results obtained in the MS can be made to other pathologic states with evidence of sympathetic activation [22, 23, 30]. This conclusion is supported by the finding that in the obese state no difference in the sympathetic overdrive can be found in the different categories of HR, which therefore is unable to identify individuals with more pronounced sympathetic overdrive [31]. The second results refers to the finding that, again in sharp contrast with we and others found in hypertension, obesity and diabetes mellitus [15, 27] plasma NE in the MS represents a sympathetic biomarker with a sensitivity similar to the one characterizing the gold standard approach for evaluating human sympathetic drive, namely microneurographic recording of MSNA [15].

A limitation of our study was represented by its retrospective nature, which allowed us, however, to analyze data collected in one of the largest database of MS patients in which different direct and indirect indices of sympathetic drive have been examined. In addition the results we obtained refer to untreated patients with the MS and thus cannot be safely extrapolated to conditions in which lifestyle measures, pharmacological approaches or surgical bariatric interventions aimed at counteracting the specific abnormalities clustering in the MS are applied.

In conclusion, our study shows that in patients with the MS HR values greater than or equal to 80 beats/min are associated with an increased sympathetic activation, both when assessed by direct recording of MSNA and when evaluated as plasma NE concentrations. This means that when in current clinical practice we detect in MS patients resting clinical HR values ≥ 80 beats/min we should be aware that an activation of sympathetic cardiovascular influences is present and that an appropriate non-pharmacological or pharmacological intervention capable to counteract the unfavorable cardiometabolic effects of such over activity should be initiated.

## Data Availability

The dataset analyzed during the current study are not publicly available but are available from the corresponding author on reasonable request.

## Abbreviations

MS: Metabolic syndrome
HR: Heart rate
NE: Plasma norepinephrine
BP: Blood pressure
MSNA: Muscle sympathetic nerve traffic

## References

[1] Brunner EJ, Hemingway H, Walker BR, Page M, Clarke P, Juneja M (2002). Adrenocortical, autonomic and inflammatory causes of the metabolic syndrome: nested case–control study. Circulation.

[2] Schlaich M, Straznicky N, Lambert F, Lambert G (2015). Metabolic syndrome: a sympathetic disease?. Lancet Diabetes Endocrinol.

[3] Quarti-Trevano F, Dell’Oro R, Biffi A, Seravalle G, Corrao G, Mancia G, Grassi G (2020). Sympathetic overdrive in the metabolic syndrome: meta-analysis of published studies. J Hypertens.

[4] Lee ZS, Critchley JA, Tomlinson B, Young RP, Thomas GN, Cockram CS (2001). Urinary epinephrine and norepinephrine interrelations with obesity, insulin and the metabolic syndrome in Hong Kong Chinese. Metabolism.

[5] Nestel PJ, Khan AA, Straznicky NE, Mellett NA, Jayawardana K, Mundra PA (2017). Markers of sympathetic nervous system activity associate with complex plasma lipids in metabolic syndrome subjects. Atherosclerosis.

[6] Rodriguez-Colon SM, He F, Bixler EO, Fernandez-Mendoza J, Vgontazas AN, Calhoun S (2015). Metabolic syndrome burden in apparently healthy adolescents is adversely associated with cardiac autonomic modulation—Penn state children cohort. Metabolism.

[7] Straznicky NE, Grima MT, Sari CI, Eikelis N, Lambert EA, Nestel PJ (2012). Neuroadrenergic dysfunction along the diabetes continuum: a comparative study in obese metabolic syndrome subjects. Diabetes.

[8] Straznicky NE, Grima MT, Sari CI, Karapanagiotidis S, Wong C, Eikelis N (2013). The relation of glucose metabolism to left ventricular mass and function and sympathetic nervous system activity in obesity with metabolic syndrome. J Clin Endocrinol Metab.

[9] Hugget RJ, Burns J, Mackintosh AF, Mary DA (2004). Sympathetic neural activation in nondiabetic metabolic syndrome and its further augmentation by hypertension. Hypertension.

[10] Grassi G, Dell’Oro R, Quarti Trevano F, Scopelliti F, Seravalle G, Paleari F (2005). Neuroadrenergic and reflex abnormalities in patients with metabolic syndrome. Diabetologia.

[11] Palatini P (2013). Heart rate and the cardiometabolic risk. Curr Hypertens Rep.

[12] Wu X, Du R, Hu C, Cheng D, Ma L, Li M (2018). Resting heart rate is associated with metabolic syndrome and predicted 10-year risk of cardiovascualr disease: across-sectional study. J Diabetes.

[13] Williams B, Mancia G, Spiering W, Agabiti Rosei E, Azizi M, Burnier M (2018). 2018 ESC/ESH guidelines for the management of arterial hypertension: the task force for the management of arterial hypertension of the European Society of Cardiology and the European Society of Hypertension. Eur Heart J.

[14] Casagrande S, Cowie CC, Sosenko JM, Mizokami-Stout K, Boulton AJM, Pop-Busui R (2020). The association between heart rate and glycemic status in the National Health and Nutrition Examination Surveys. J Clin Endocrinol Metab.

[15] Grassi G (2009). Assessment of sympathetic cardiovascular drive in human hypertension: achievements and perspectives. Hypertension.

[16] Grundy SM, Cleeman JI, Daniels SR, Donato KA, Eckel RH, Franklin BA (2005). Diagnosis and management of the metabolic syndrome: an American Heart Association/National Heart, Lung, and Blood Institute Scientific Statement. Circulation.

[17] Matthews DR, Hosker JP, Rudenski AS, Naylor BA, Treacher DF, Turner RC (1985). Homeostasis model assessment: insulin resistance and beta-cell function from fasting plasma glucose and insulin concentrations in man. Diabetologia.

[18] Javidan S, Cwik MJ (1996). Determination of catecholamines in human plasma by HPLC with electrochemical detection. J Liq Chromatogr Relat Technol.

[19] Schiller NB, Shah PM, Crawford M, DeMaria A, Devereux R, Feigenbaum H (1989). Recommendations for quantitation of the left ventricle by two-dimensional echocardiography. American Society of Echocardiography Committee on standards, subcommittee on quantitation of two-dimensional echocardiograms. J Am Soc Echocardiogr.

[20] Park SH, Shub C, Nobrega TP, Bailey KR, Seward JB (1996). Two-dimensional echocardiographic calculation of left ventricular mass as recommended by the American Society of Echocardiography: correlation with autopsy and M-mode echocardiography. J Am Soc Echocardiogr.

[21] Grassi G, Seravalle G, Cattaneo BM, Lanfranchi A, Vailati S, Giannattasio C (1995). Sympathetic activation and loss of reflex sympathetic control in mild congestive heart failure. Circulation.

[22] Grassi G, Quarti-Trevano F, Seravalle G, Dell’Oro R, Facchetti R, Mancia G (2020). Association between the European Society of Cardiology/European Society of Hypertension heart rate thresholds for cardiovascular risk and neuroadrenergic markers. Hypertension.

[23] Grassi G, Seravalle G, Vanoli J, Facchetti R, Spaziani D, Mancia G (2022). Relationships between sympathetic markers and heart rate tresholds for cardiovascular risk in chronic heart failure. Clin Res Cardiol.

[24] Cohn J, Levine T, Olivari MT, Garberg V, Lura D, Francis GS, Simon AB, Rector T (1984). Plasma norepinephrine as a guide to prognosis in patients with congestive heart failure. N Engl J Med.

[25] Zoccali C, Mallamaci F, Parlongo S, Cutrupi S, Benedetto FA, Tripepi G (2002). Plasma norepinephrine predicts survival and incident cardiovascular events in patients with end-stage renal disease. Circulation.

[26] Sander D, Winbeck K, Klingelhofer J, Etgen T, Conrad B (2001). Prognostic relevance of pathological sympathetic activation after acute thromboembolic stroke. Neurology.

[27] Esler M, Rumantir M, Wiesner G, Kaye D, Hastings J, Lambert G (2001). Sympathetic nervous system and insulin resistance:from obesity to diabetes. Am J Hypertens.

[28] Sharma AM, Chetty VT (2005). Obesity, hypertension and insulin resistance. Acta Diabetol.

[29] Yu Y, Hu L, Xu Y, Wu S, Chen Y (2020). Impact of blood glucose control on sympathetic and vagus nerve functional status in patients with type 2 diabetes mellitus. Acta Diabetol.

[30] Grassi G, Fowler B, Scali B, Rossi F, Motto E, Pieruzzi F, et al (2022) Sympathetic activation and heart rate tresholds for cardiovascular risk in chronic kidney disease. In: Abstract submitted to the 31st European meeting on hypertension and cardiovascular protection, Athens 2022, June 17–20, J Hypertens 40 (suppl 1) **(in press)** 10.1097/HJH.0000000000003179 PMC10860883 35792492

[31] Seravalle G, Facchetti R, Cappellini C, Annaloro A, Gelfi E, Grassi G (2022) Elevated heart rate as sympathetic biomarker in human obesity. In: Abstract submitted to the 31st European meeting on hypertension and cardiovascular protection, Athens 2022, June 17–20, J Hypertens 40 (suppl 1) **(in press)** 10.1016/j.numecd.2022.07.011 35970685

